# Breastfeeding predicts blood mitochondrial DNA content in adolescents

**DOI:** 10.1038/s41598-019-57276-z

**Published:** 2020-01-15

**Authors:** Charlotte Cosemans, Tim S. Nawrot, Bram G. Janssen, Annette Vriens, Karen Smeets, Willy Baeyens, Liesbeth Bruckers, Elly Den Hond, Ilse Loots, Vera Nelen, Nicolas Van Larebeke, Greet Schoeters, Dries Martens, Michelle Plusquin

**Affiliations:** 1 Centre for Environmental Sciences, Hasselt University, Diepenbeek, Belgium; 2 School of Public Health, Occupational & Environmental Medicine, Leuven University, Leuven, Belgium; 3 Department of Analytical and Environmental Chemistry, Vrije Universiteit Brussel, Brussels, Belgium; 4 Interuniversity Institute for Biostatistics and Statistical Bioinformatics, Hasselt University, Hasselt, Belgium; 5 Provincial Institute for Hygiene, Antwerp, Belgium; 6 Faculty of Social Sciences and IMDO-Institute, University of Antwerp, Antwerp, Belgium; 7 Department of Radiotherapy and Experimental Cancerology, Ghent University, Ghent, Belgium; 8 Department of Analytical, Environmental and Geo-Chemistry, Vrije Universiteit Brussel, Brussels, Belgium; 9 Environmental Risk and Health, Flemish Institute for Technological Research (VITO), Mol, Belgium

**Keywords:** Nutrition, Epidemiology

## Abstract

Nutrition during early childhood is linked to metabolic programming. We hypothesized that breastfeeding has long-term consequences on the energy metabolism exemplified by mitochondrial DNA (mtDNA). As part of the third cycle of the Flemish Environment and Health Study (FLEHSIII) cohort, 303 adolescents aged 14–15 years were included. We associated breastfeeding and blood mtDNA content 14–15 years later while adjusting for confounding variables. Compared with non-breastfed adolescents, mtDNA content was 23.1% (95%CI: 4.4–45.2; p = 0.013) higher in breastfed adolescents. Being breastfed for 1–10 weeks, 11–20 weeks, and >20 weeks, was associated with a higher mtDNA content of respectively 16.0% (95%CI: −7.1–44.9; p = 0.191), 23.5% (95%CI: 0.8–51.3; p = 0.042), and 31.5% (95%CI: 4.3–65.7; p = 0.021). Our study showed a positive association between breastfeeding and mtDNA content in adolescents which gradually increased with longer periods of breastfeeding. Higher mtDNA content may be an underlying mechanism of the beneficial effects of breastfeeding on children’s metabolism.

## Introduction

Exclusive breastfeeding (i.e. not formula-feeding) for six months is recommended by the World Health Organization (WHO) for optimal growth, development, and health of the child^1^. Breast milk is known to have many benefits for the child’s health, like strengthening their immune system and it has a positive effect on brain development and cognitive function. Studies on the effect of breastfeeding stated a decline in infection rates in childhood^2^, an improvement in white matter development^3^, a better psychomotor development during the first year of life^4^, and higher cognitive development scores^5^. Breastfeeding may, in addition, be associated with a lower risk of type 2 diabetes mellitus^6^ and has a protective effect against obesity^7^. The potential mechanisms through which breastfeeding can have positive effects on children’s development may be due to the composition of breast milk (i.e. presence of nutrients and absence of preservatives), the optimal supply of breast milk, or the improved bond between mothers and their child^8^. However, the exact cellular and molecular mechanisms through which breastfeeding exerts its positive effects are still unknown.

Mitochondria are intracellular organelles responsible for energy production by producing adenosine triphosphate (ATP), a substrate required for metabolism. Every cell contains various mitochondria, each with multiple copies of mitochondrial DNA (mtDNA)^9^. Decreased mitochondrial function can cause impaired cellular functions and give rise to a variety of human diseases such as cardiovascular diseases^10^, cancer^11^, diabetes mellitus and metabolic syndrome^12,13^, autoimmune diseases^14,15^, and neurodegenerative and -behavioral diseases^16–18^. In addition, placental mtDNA content was positively associated with neurocognition in children^19^. Impaired mitochondrial function also plays a role in obesity-related^7^ and cardiovascular diseases^20^. Mitochondrial function can be altered by environmental or life style factors, including nutrient supply. For example, diverse dietary fat sources change mitochondrial function in different ways. In contrast to saturated fatty acids, omega 3 polyunsaturated fatty acids, which are also present in breast milk^21^, improved mitochondrial function and reduced reactive oxygen species (ROS) production^22^.

mtDNA content can be an indicator of mitochondrial (dys)function^23^. A lower mtDNA copy number is a potential biomarker for type 2 diabetes mellitus^24^, while an increased mtDNA copy number is linked with a lower risk of metabolic syndrome in adults^25^. Furthermore, mtDNA content has been positively associated with metabolic hormones such as leptin and insulin in early life^26,27^. In addition, a reduced mtDNA copy number has also been associated with aging^28–30^ and has been suggested as a biomarker for age-related neurodegenerative diseases, such as Parkinson’s disease^31^.

It is hypothesized that early-life conditions contribute to the predisposition for health or disease later in life. Breastfeeding might establish mitochondrial function in later life stages, but no data are available so far. Here we focus on adolescents and study if breastfeeding as an infant is associated with mtDNA content, an indicator of mitochondrial function.

## Results

### Population characteristics

The mean age of all adolescents was 14.9 ± 0.6 years and they had a mean BMI of 19.5 ± 3.1 kg/m². 52.5% of the participants were girls. Of these adolescents, 99.3% were Caucasian. The majority of the adolescents never smoked (89.1%) and were exposed to passive smoking less than once per week (60.7%). The majority of the mothers were between 25–30 years old at delivery (48.8%) and had a high household SES (50.0%). In total, 11.6% of the mothers reported to smoke and 19.1% reported alcohol consumption during pregnancy. 60.4% of the mothers breastfed their child with an average of 11.6 ± 8.6 weeks of exclusively breastfeeding (Supplementary Table S1). The age of the adolescents (p = 0.05), the SES of the household (p < 0.001), and the smoking status of the mother during pregnancy (p = 0.01) differed between breastfed and non-breastfed groups (Table 1). Adolescents were grouped based on the duration of breastfeeding **(**Table 1**)**. Without adjusting for confounding variables, mtDNA content was higher when longer breastfed, but this was not significant (*p* = 0.13) **(**Supplementary Fig. S1).

**Table 1:** Study population characteristics, subdivided for breastfeeding.

Characteristic	Breastfed (n = 183)	Without breastfeeding (n = 120)	*p*-value
*Adolescents*	*Mean* ± *SD or n (%)*	*Mean* ± *SD or n (%)*	
Age (years)	14.9 ± 0.6	15.0 ± 0.6	0.05
Sex			0.19
Female	90 (49.2)	69 (57.5)	
BMI	19.6 ± 2.9	19.4 ± 3.3	0.77
Smoking			0.71
Never	165 (90.1)	105 (87.5)	
Occasional	12 (6.6)	9 (7.5)	
Daily	6 (3.3)	6 (5.0)	
Passive smoking			0.47
Never	42 (23.0)	22 (18.3)	
<1 per week	111 (60.6)	73 (60.9)	
>1 per week	30 (16.4)	25 (20.8)	
Alcohol			0.31
Never	105 (57.4)	58 (48.3)	
<monthly	37 (20.2)	34 (28.3)	
<weekly	32 (17.5)	20 (16.7)	
Weekly	9 (4.9)	8 (6.7)	
Season of sampling			0.71
Winter	19 (10.4)	15 (12.5)	
Spring	92 (50.3)	57 (47.5)	
Summer	4 (2.2)	5 (4.2)	
Autumn	68 (37.1)	43 (35.8)	
mtDNA content	1.20 ± 0.65	1.08 ± 0.55	0.11
*Maternal*	*Mean* ± *SD or n (%)*	*Mean* ± *SD or n (%)*	
Age at delivery			0.25
≤25 years	30 (16.4)	29 (24.2)	
25–30 years	93 (50.8)	55 (45.8)	
>30 years	60 (32.8)	36 (30.0)	
Smoking during pregnancy			0.01
Yes	13 (7.1)	22 (18.3)	
Alcohol during pregnancy			0.46
Yes	38 (20.8)	20 (16.7)	
High blood pressure			0.52
Yes	4 (2.2)	5 (4.2)	
Pre-term delivery			0.81
Yes	16 (8.7)	12 (10.3)	
Total weeks of breastfeeding			<0.001
0 weeks	—	120 (100.0)	
1–10 weeks	55 (30.0)	—	
11–20 weeks	77 (42.1)	—	
>20 weeks	51 (27.9)	—	
Socioeconomic status household			<0.001
Low	17 (9.3)	14 (11.7)	
Middle	55 (30.0)	64 (53.3)	
High	111 (60.7)	42 (35.0)	

### Breastfeeding in association with mtDNA content

After adjustment for BMI, sex, age, SES household, smoking, passive smoking, alcohol consumption of the adolescent, season, smoking of the mother during pregnancy, and age of mother at delivery, breastfeeding was positively associated with mtDNA content. Receiving breastfeeding in early life was associated with a 21.3% (95% CI: 2.9 to 42.9; p = 0.02) higher mtDNA content at adolescent age compared with adolescents who did not receive breastfeeding (Table 2, Supplementary Table S2). When additionally adjusted for alcohol consumption of the mother during pregnancy, high blood pressure of the mother, and pre-term birth, breastfeeding was associated with a 23.1% (95% CI: 4.4 to 45.2; p = 0.01) higher mtDNA content.

**Table 2:** The association between breastfeeding and relative mitochondrial DNA content compared to non-breastfed adolescents.

	n	% Difference (95% CI)	*p*-value
**Models:**			
Model 1	303	21.3% (2.9 to 42.9)	0.02
Model 2	303	23.1% (4.4 to 45.2)	0.01
**Sensitivity analysis (Model 2):**			
Excluding mothers that smoked during pregnancy ^(1),$^	268	22.6% (2.3 to 47.0)	0.03
Excluding smokers during adolescence ^(2),$^	270	26.9% (6.0 to 52.0)	0.01
Combination of ^(1)^ and ^(2)^	244	26.2% (4.0 to 53.3)	0.02
Only females	159	14.7% (−10.8 to 47.6)	0.28
Only males	144	27.8% (3.0 to 58.6)	0.03
Additionally adjusted for leukocyte amount	303	23.1% (4.4 to 45.2)	0.01
Additionally adjusted for region	303	26.1% (7.6 to 48.1)	0.004
Additionally adjusted for physical activity	300	22.8% (3.9 to 44.9)	0.02
Additionally adjusted for birthweight	295	23.9% (4.8 to 46.4)	0.01
Additionally adjusted for genital development males	140	26.1% (1.4 to 56.8)	0.04
Additionally adjusted for breast development females	159	17.0% (−9.9 to 51.7)	0.24

Model 1: adjusted for BMI, sex, age, SES household, smoking, passive smoking, alcohol consumption of the adolescent, season, smoking of the mother during pregnancy, and age of mother at delivery. Model 2: model 1 additionally adjusted for alcohol consumption of the mother during pregnancy, high blood pressure mother, and pre-term birth. Estimates were presented as % difference (95% CI) in mtDNA content for adolescents that received breastfeeding compared to non-breastfed adolescents. ^$^Both occasional and daily smokers were excluded.

**Figure 1:**
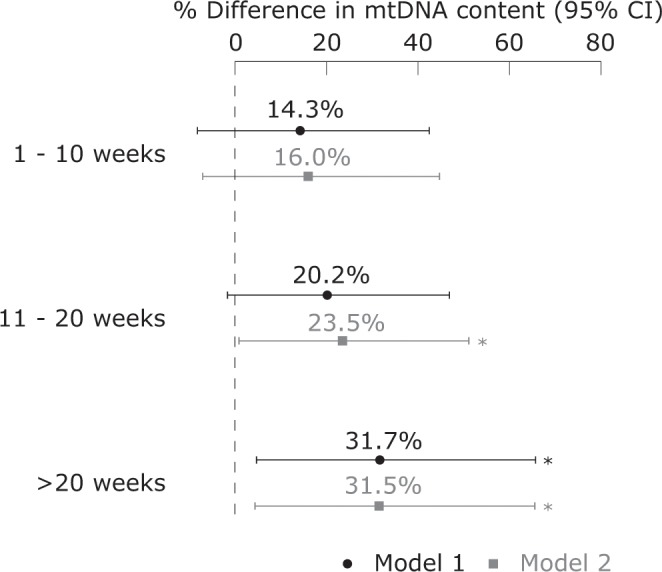
The association between weeks of breastfeeding and relative mitochondrial DNA content compared to non-breastfed adolescents. Model 1: adjusted for BMI, sex, age, SES household, smoking, passive smoking, alcohol consumption of the adolescent, season, smoking of the mother during pregnancy, and age of mother at delivery. Model 2: additionally adjusted for alcohol consumption of the mother during pregnancy, high blood pressure mother, and pre-term birth. *p < 0.05

Part of the adolescents did not receive breastfeeding (n = 120) while respectively 55, 77, and 51 adolescents received 1–10 weeks, 11–20 weeks, and >20 weeks of breastfeeding during infancy. The association between mtDNA content and breastfeeding is stronger when infants were longer breastfed compared to non-breastfed adolescents (Fig. 1). When infants were breastfed for 1–10 weeks, 11–20 weeks, and more than 20 weeks, mtDNA content was increased with 16.0% (95% CI: −7.1 to 44.9; p = 0.19), 23.5% (95% CI: 0.8 to 51.3; p = 0.04), and 31.5% (95% CI: 4.3 to 65.7; p = 0.02), respectively, compared with the non-breastfed group, after adjustment for BMI, sex, age, SES household, smoking, passive smoking, alcohol consumption of the adolescent, season, smoking and alcohol consumption during pregnancy, high blood pressure mother, age of mother at delivery, and pre-term birth.

In sensitivity analyses (Table 2), we showed that exclusion of mothers who smoked during pregnancy and adolescents who smoked, as well as adjusting for leukocyte amount, region, physical activity, birthweight, genital development in males, and breast development in girls did not affect our results. In a stratified analysis per sex, we found that the effect of breastfeeding on mtDNA content was only significant in boys (p = 0.03) not in girls (p = 0.28). However, the interaction of the sex by breastfeeding on blood mtDNA content was not significant (p = 0.50). We additionally adjusted for several pregnancy complications: intrauterine growth restriction, preeclampsia, and gestational diabetes mellitus. However, this did not affect the estimates (from 23.1% to 22.9%, 22.0%, and 24.6%, respectively) nor p-values (from 0.01 to 0.02, 0.01, and 0.01, respectively) of our findings.

## Discussion

In this study, we observed a positive association between breastfeeding during infancy on mtDNA content at adolescent age. The longer the adolescent was postnatally breastfed, the higher the blood mtDNA content was at adolescent age, suggesting a duration-dependent effect of breastfeeding. The effect of breastfeeding was also dose-dependent, with longer duration and exclusivity increasing its effects^32^. The WHO recommended to further breastfed infants up to two years^33^, as children’s health and development continued to benefit from the provision of breast milk^34^. To our knowledge, no other study previously described this association and it might entail an important mechanism to explain the positive effects of breastfeeding on the energy metabolism^35–37^. There are several mechanisms through which breastfeeding can exert its positive effects on mtDNA, such as the energy metabolism, metabolic hormones, antioxidant compounds, and neurocognitive development, elucidated below.

Breastfeeding has a protective effect against obesity^7^. In contrast to formula feeding, breastfeeding is mainly on demand and less prone to overfeeding. Mothers in a lower SES of the household were less likely to breastfeed their child, which has been described by several other studies^38–41^. Although the epidemiologic evidence about the relationship between adiposity and mtDNA content was limited, an inverse association of mtDNA content with the visceral fat area and waist circumference has been reported^42–44^ and BMI has been linked with less mtDNA content in middle aged adults^45^. Civitarese *et al*.^46^ demonstrated, using a clinical trial, that patients who consumed 25% less calories than normal (i.e. caloric restricted patients), showed a lower amount of mtDNA damage, a higher mtDNA content, and increased levels of antioxidant enzymes. In addition, an experimental animal study in young and adult rats demonstrated that neonatal overfeeding caused dysfunction of the mitochondrial respiratory chain complex in the heart^47^. Caloric restriction might enhance mitochondrial functioning and expand their longevity via reducing oxidative stress^30^.

A positive association between metabolic hormones (i.e. leptin and insulin) and mtDNA content in early life has been reported^26,27^. Breast milk contains the hormone leptin, which may reduce psychosocial stress in infants^48^. Leptin is involved in energy homeostasis and could regulate the energy balance in child- and adulthood^35^. When administering leptin to leptin-deficient children, their energy intake and fat mass decreased, while lean body mass was unaffected^49^. In an *in vitro* study, leptin exposure increased PGC-1α expression, an important factor in mitochondrial biogenesis, as well as decreased the levels of H_2_O_2_. These data suggests that leptin plays a role in mitochondrial biogenesis by avoiding the production of ROS^50^.

Lower blood glucose and serum insulin concentrations in childhood and lower insulin levels in later life have been associated with breastfeeding. Breastfeeding has as such been associated with a decreased risk of type 2 diabetes mellitus^6^. The function of mitochondria is closely related to insulin secretion and possibly insulin activity^51,52^. mtDNA content may be linked with type 2 diabetes mellitus and can serve as an indicator of insulin sensitivity^53^. Lee *et al*.^54^ reported that blood mtDNA content was 25–35% lower in type 2 diabetes mellitus cases, compared with healthy individuals. Also in pre-diabetic subjects who progressed to type 2 diabetes mellitus within two years, a lower blood mtDNA content was reported.

Mitochondria play an important role in the energy metabolism and maintaining normal physiology in the cell. A loss in mitochondrial function is induced by aging and several studies reported a decline in mtDNA content with aging^28–30,55^. The study of Mengel-From *et al*.^56^ supported these findings by demonstrating that mtDNA copy number declines while ageing, with 5.4 mtDNA copies less every 10 years from the age of 48. In addition, age-related mitochondrial dysfunction has been reported to contribute to insulin resistance, a major factor in type 2 diabetes mellitus, in the elderly^57^. In a larger study with approximately 1000 subjects, mtDNA copy numbers were significantly reduced with age, but only in males^58^. Although the interaction of the sex by breastfeeding on blood mtDNA content was not significant, stratified analysis showed only significant associations in adolescent boys. Sex steroid hormones are able to regulate mitochondrial function. Moreover, mitochondria play an important role in the biosynthesis of these hormones^59^. Experimental studies in rats showed sex-related differences in mitochondrial function. Remarkably, male rats were more susceptible to mtDNA damage induced by ROS^60^. In contrast to our higher mtDNA content in boys, population based studies in adults reported lower peripheral blood mtDNA content in men compared with women^61,62^. Baseline sex-related differences in mtDNA content might explain potential higher susceptibility in mtDNA content changes in boys compared with girls. In addition, Lucas *et al*.^63^ observed that infants responded differently to their early nutritional environment dependent on their sex. Experimental studies reported that breast milk composition was determined by infant’s sex^64–66^. A better understanding of the mechanisms driving sex-dependent milk synthesis is needed to further investigate the potential different nutritional and hormonal intakes in males and females, and their possible effect on blood mtDNA content in later life.

Our findings showed a duration effect for the positive association between being breastfed and blood mtDNA content, and in a stratified analysis we observed this from 11 weeks of breastfeeding onwards. This is in concordance with a multitude of studies describing beneficial effects of longer breastfeeding^7,67^. Although many studies focused on long-term breastfeeding (i.e. for at least six months), our study demonstrated that also shorter term breastfeeding has an impact at the biomolecular level, such as the mtDNA content in peripheral blood.

Melatonin, also present in human milk, is a well-known antioxidant. Due to its small size, it can reach various cellular components, in particular mitochondria. Several studies reported that melatonin had a protective role in mitochondrial homeostasis^68,69^. During pregnancy, the placenta produces melatonin, while after delivery, it is passed to the child through breastfeeding^70^, where it can exert an important role in antioxidant defence sustaining the integrity, stability, and function of mitochondrial membranes^71^. However, to elucidate the protective role of melatonin, further research is necessary.

Breastfeeding is known to have a beneficial effect on the cognitive development of the child^3–5^ and to be related to improved performance in intelligence tests^72^. Since mitochondrial dysfunction plays an important role in neurodegenerative disorders^73^, it may be possible that an increase of mtDNA content has beneficial effects on brain development. The brain is sensitive to mitochondrial protein synthesis during early postnatal development, probably due to the massive mitochondrial biogenesis occurring at that stage^74^. Maternal milk is a rich source of fatty acids and other bioactive components that are essential for brain development. In mitochondria, fatty acids serve as energy sources, molecules for post-translational modifications of proteins, membrane components and as signalling factors^75^. The study of Oddy *et al*.^76^ also reported that a shorter duration of breastfeeding was associated with increased mental health morbidity throughout a period spanning early childhood to adolescence. Future studies may elucidate the role of mtDNA in the association between breastfeeding and cognition.

The study has several limitations and strengths. As this study was part of FLEHS III, it is representative of the population living in Flanders^77^. Since it is a retrospective study and the data on breastfeeding was collected several years after the mother breastfed her child, it might suffer from a recall bias. On the other hand, the fact that there are 14 or 15 years between breastfeeding and the analysis of mtDNA content, gives an indication of the long-term effect of breastfeeding. Since the turnover rate of mtDNA is estimated to range from ~2 to 350 days^78^, these data suggest a possible metabolic alteration influencing the mitochondrial biogenesis on longer term. Wachsmuth *et al*.^55^ analysed the mtDNA copy numbers from different tissues and reported that each tissue of an individual seemed to regulate their mtDNA copy number in a tissue-related manner. Because the total number of mtDNA copies per cell varies between different tissues of the same individual, our findings are limited to peripheral blood. However, a correlation between myocardial DNA content and peripheral blood mtDNA content was reported^79^. While this study focussed on mtDNA content as a proxy for energy metabolism, also other mitochondrial (e.g. oxidative phosphorylation) or other energy-related processes (e.g. amount of fatty acids) might be involved. Mitochondrial function can be altered by environmental or life style factors, such as nutrition. Unfortunately, we did not have a food frequency questionnaire. We additionally adjusted our models for leukocyte counts, since changes in the leukocyte cell proportions might influence the mtDNA content. In addition, as stated by Hurtado-Roca *et al*.^80^, platelet counts should also be taken into account when measuring mtDNA content. However, these data were not available.

## Conclusion

To our knowledge, we were the first to show a link between breastfeeding and the mtDNA content. Our findings indicate that breastfeeding is associated with a higher blood mtDNA content in a general population of adolescents. This increase gradually enhances with longer breastfeeding. Our results may contribute to the ‘Developmental Origins of Health and Disease’ hypothesis (DOHaD) that states that health or diseases may find their origin in early life^81^. Nevertheless, it remains unclear through which mechanism breastfeeding has an effect on mtDNA content in later life and more studies are needed to elaborate on the role of breastfeeding on later life function of mitochondria.

## Materials and Methods

### Study population

This study was part of the third cycle of the Flemish Environment and Health study (FLEHS), which recruited 355 adolescents aged 14–15 years from the general population of Flanders, Belgium (reference population) (n = 196) and from the industrial area in Ghent, Belgium (canal zone) (n = 159). After excluding 52 subjects due to missing data (mtDNA, n = 19; breastfeeding, n = 3; weeks of breastfeeding, n = 7; other variables, n = 23), data were analysed for 164 adolescents from the reference population and 139 adolescents from the industrial area.

A stratified clustered multi-stage design was used to select participants within schools. Recruitment was spread over one year (2013) with no recruitment of adolescents during examination and summer holidays^77^.

The medical ethical committee of Antwerp University and University Hospital of Antwerp approved the study. Informed consent was obtained from a parent and/or legal guardian for study participation. This study has been carried out according to the Helsinki declaration.

### Data and sample collection

The use of questionnaires and sampling has been described elsewhere^82^. Briefly, all participants provided a questionnaire completed by both the adolescent and the parents. Mothers filled out a questionnaire addressing their health status during pregnancy (e.g. age of the mother at delivery, complications such as high blood pressure during pregnancy and pre-term birth (<37 weeks)), their lifestyle during pregnancy (e.g. smoking and alcohol consumption), the first years of life of their child (e.g. birthweight, breastfeeding and exposure to passive smoking), general information of their child (e.g. BMI (kg/m²), age, sex, physical activity (never or rarely/1–2 times per week/≥3 times per week)), and the socio-economic status of their household (household SES), coded low (no diploma or primary school), middle (high school) or high (college or university) based on the highest education. Puberty development in girls was assessed by breast development and pubis hair, following the method of Marshall & Tanner^83^ and was scored between 1 to 5. Puberty development in boys was assessed by genital development and pubis hair, according to the method of Marshall & Tanner^84^ and was scored between 1 to 5. Breastfeeding was coded yes/no based on the questionnaires as well as the number of weeks of breastfeeding (0, 1–10, 11–20, and >20 weeks). Additionally, a questionnaire with possibly sensitive questions on smoking (never/occasional/daily) and alcohol (never/<monthly/<weekly/weekly) was answered by the adolescents without supervision by the parents. A 35 mL blood sample was collected during the fieldwork in EDTA Vacutainer Blood Collection Tubes (Becton Dickinson) and stored at −80 °C until further use.

### Measurement of mitochondrial DNA content

Mitochondrial DNA content was measured as described elsewhere^27^. Briefly, DNA was isolated from whole blood using the QIAamp DNA mini kit (Qiagen). The relative amount of mtDNA was measured by determining the ratio of two mitochondrial gene copy numbers (MTF3212/R3319 and MT-ND1) to a single-copy nuclear control gene (RPLP0) using the 7900HT Fast Real-Time PCR System (Applied Biosystems). The full protocol is available in the supplemental information.

### Statistical analysis

Data management and statistical analysis were done using RStudio software (version 1.1.456). mtDNA content was transformed with the natural logarithm to normalize its distribution. Continuous variables are presented as means ± standard deviation (SD). Categorical variables are presented as numbers (frequency in percentage). Age of the mother at delivery, high blood pressure during pregnancy, pre-term birth, smoking and alcohol consumption during pregnancy, breastfeeding, passive smoking (i.e. second hand smoking not at home), sex, household SES, weeks of breastfeeding, smoking and alcohol consumption of the adolescent, season, physical activity, breast development in girls, genital development in boys, and region (reference or industrial area population) were categorical variables. BMI, age, birthweight, and leukocyte amount (amount/µl) were continuous variables. The study population characteristics between breastfed and non-breastfed adolescents were compared using a Student’s t-test for continuous variables and a Pearson’s chi-squared test for categorical variables.

The association between blood mtDNA content of 14–15 year adolescents and breastfeeding was explored using multiple linear regression. Models were adjusted for the following *a priori* selected covariates: adolescents’ age, sex, smoking, passive smoking, alcohol consumption, season of sampling, region, as well as maternal smoking behaviour, alcohol consumption during pregnancy, household SES, and leukocyte amount. Adolescents’ BMI was forced into the model regardless of the *p-*value. In a secondary analysis, we grouped the study population based on weeks of breastfeeding (0, 1–10, 11–20, and >20 weeks) to explore a possible duration-dependent effect of breastfeeding on blood mtDNA content. In a sensitivity analysis, we examined the association between breastfeeding and blood mtDNA content when excluding adolescents who smoked, mothers who smoked during pregnancy, adjusted for leukocyte amount, region, physical activity, birthweight genital development in males, and breast development in girls, and stratified by sex. Estimates were provided as a % difference (95% CI).

## Supplementary information

Supplementary Information.

## Data Availability

Extra information on methods, data and protocols are available upon request.
