# Supplementary material for: Breastfeeding predicts blood mitochondrial DNA content in adolescents
Source: Sci Rep. 2020 Jan 15;10:387. doi: 10.1038/s41598-019-57276-z (PMC6962168; doi:10.1038/s41598-019-57276-z)
Supplement: Supplementary file 1 — Supplementary Information. [file 41598_2019_57276_MOESM1_ESM.pdf]

Breastfeeding predicts blood mitochondrial DNA content in adolescents

Cosemans Charlotte[1], Nawrot Tim S[1,2], Janssen Bram G[1], Vriens Annette[1], Smeets Karen[1], Baeyens Willy[3], Bruckers Liesbeth[4], Den Hond Elly[5], Loots Ilse[6], Nelen Vera[5], Van Larebeke Nicolas[7,8], Schoeters Greet[9], Martens Dries[1], Plusquin Michelle[1*].

[1] Centre for Environmental Sciences, Hasselt University, Diepenbeek, Belgium

[2] School of Public Health, Occupational & Environmental Medicine, Leuven University, Leuven, Belgium

[3] Department of Analytical and Environmental Chemistry, Vrije Universiteit Brussel, Brussels, Belgium

[4] Interuniversity Institute for Biostatistics and Statistical Bioinformatics, Hasselt University, Hasselt, Belgium

[5] Provincial Institute for Hygiene, Antwerp, Belgium

[6] Faculty of Social Sciences and IMDO-Institute, University of Antwerp, Antwerp, Belgium

[7] Department of Radiotherapy and Experimental Cancerology, Ghent University, Ghent, Belgium

[8] Department of Analytical, Environmental and Geo-Chemistry, Vrije Universiteit Brussel, Brussels, Belgium

[9] Environmental Risk and Health, Flemish Institute for Technological Research (VITO), Mol, Belgium

* Corresponding author: michelle.plusquin@uhasselt.be

SUPPLEMENTAL INFORMATION

MEASUREMENT OF MITOCHONDRIAL DNA CONTENT

DNA was isolated from whole blood using the QIAamp DNA mini kit (Qiagen). The relative amount of mtDNA was measured by determining the ratio of two mitochondrial gene copy numbers (MTF3212/R3319 and MT-ND1) to a single-copy nuclear control gene (RPLP0) using a real-time quantitative polymerase chain reaction (qPCR). qPCR reactions were carried out in triplicate on a 384-well plate on the 7900HT Fast Real-Time PCR System (Applied Biosystems) in a 10 µl volume containing: 5 µl Fast SYBR Green (Applied Biosystems) mastermix, 0.3 µl of forward and reverse primers (300 nM) and 1.9 µl RNAse-free water and 6 ng DNA diluted in 2.5 µl RNAse-free water. Primer sequences for mitochondrial genes are reported elsewhere (1). Six interrun calibrators and no-template controls were included in each qPCR run. The thermal cycling profile for the three targets was 10 min at 95 °C for activation of the polymerase enzyme and initial denaturation, followed by 40 cycles of 15 s at 94 °C for denaturation and 70 s at 58 °C for annealing and extension. After thermal cycling, the raw data were collected and processed using SDS 2.3 software (Applied Biosystems). The cycle quantification (Cq) values were normalized relatively to the RPLP0 gene using qBase + software (Biogazelle) taking into account the run-to-run differences (2).

SUPPLEMENTARY FIGURES/TABLES

Supplementary Table S1: Population characteristics.

**Characteristic**		**Total population**	**Study population (n=303)**
Adolescents	n	Mean ± SD or *n* (%)	Mean ± SD or *n* (%)
Age (years)	355	14.9 ± 0.6	14.9 ± 0.6
Sex	355		
Female		186 (52.4)	159 (52.5)
BMI	355	19.6 ± 3.2	19.5 ± 3.1
Smoking	351		
Never		312 (88.9)	270 (89.1)
Occasional		27 (7.7)	21 (6.9)
Daily		12 (3.4)	12 (4.0)
Passive smoking (not at home)	349		
Never		73 (20.9)	64 (21.1)
< 1 per week		209 (59.9)	184 (60.7)
> 1 per week		67 (19.2)	55 (18.2)
Alcohol	352		
Never		187 (53.1)	163 (53.8)
< monthly		84 (23.9)	71 (23.4)
< weekly		62 (17.6)	52 (17.2)
Weekly		19 (5.4)	17 (5.6)
Season of sampling	355		
Winter		43 (12.1)	34 (11.2)
Spring		166 (46.8)	149 (49.2)
Summer		10 (2.8)	9 (3.0)
Autumn		136 (38.3)	111 (36.6)
mtDNA content		1.16 ± 0.6	1.15 ± 0.6
Maternal			
Age at delivery	349		
≤ 25 years		66 (18.9)	59 (19.5)
25 – 30 years		176 (50.4)	148 (48.8)
> 30 years		107 (30.7)	96 (31.7)
Smoking during pregnancy	349		

Yes		41 (11.7)	35 (11.6)
Alcohol during pregnancy	352		
Yes		64 (18.2)	58 (19.1)
Breastfeeding	352		
Yes		207 (58.8)	183 (60.4)
Total weeks of breastfeeding	348		
0 weeks		145 (41.7)	120 (39.6)
1 – 10 weeks		64 (18.4)	55 (18.2)
11 – 20 weeks		84 (24.1)	77 (25.4)
> 20 weeks		55 (15.8)	51 (16.8)
Socioeconomic status household	343		
Low		36 (10.5)	31 (10.2)
Middle		135 (39.4)	119 (39.3)
High		172 (50.1)	153 (50.5)

[$] *n* = 173

Supplementary Table S2: Full results of multiple linear regression models.

	Model 1		Model 2	
	% Difference (95% CI)	***p-value***	***% Difference (95% CI)***	***p-value***
Breastfeeding	21.3% (2.9 to 42.9)	0.02	23.1% (4.4 to 45.2)	0.01
BMI	0.6% (-2.1 to 3.4)	0.67	0.6% (-2.1 to 3.3)	0.69
Sex (female)	-15.1% (-27.5 to -0.6)	0.04	-17.0% (-29.2 to -2.6)	0.02
Age	26.2% (7.1 to 48.6)	0.01	29.8% (10.1 to 53.0)	0.002
SES household (middle)	22.1% (-7.5 to 61.1)	0.16	22.5% (-7.1 to 61.8)	0.15
SES household (high)	2.7% (-22.7 to 36.5)	0.85	4.6% (-21.3 to 39.1)	0.75
Smoking (occasional)	-5.2% (-31.4 to 31.3)	0.75	-11.2% (-36.0 to 23.4)	0.48
Smoking (daily)	-13.7% (-45.3 to 36.3)	0.53	-6.7% (-41.3 to 48.4)	0.77
Passive smoking (rarely)	16.5% (-4.1 to 41.5)	0.12	17.4% (-3.7 to 43.0)	0.11
Passive smoking (>1/week)	11.9% (-14.2 to 45.8)	0.41	15.7% (-11.4 to 51.1)	0.28
Alcohol consumption adolescent (<monthly)	-13.1% (-28.8 to 6.2)	0.17	-14.4% (-30.0 to 4.8)	0.13

Alcohol consumption adolescent (<weekly)	-26.3% (-42.1 to -6.3)	0.01	-28.4% (-43.8 to -8.9)	0.007
Alcohol consumption adolescent (weekly)	-10.3% (-39.0 to 31.8)	0.58	-14.6% (-42.4 to 26.5)	0.43
Season (spring)	-22.4% (-40.7 to 1.7)	0.07	-19.0% (-38.3 to 6.3)	0.13
Season (summer)	33.4% (-20.1 to 122.8)	0.27	51.4% (-11.6 to 159.1)	0.13
Season (autumn)	-13.0% (-33.6 to 13.9)	0.31	-10.4% (-31.6 to 17.5)	0.43
Smoking of mother during pregnancy	23.0% (-5.1 to 59.2)	0.12	31.7% (1.0 to 71.4)	0.04
Age of mother at delivery (25-30 years)	-1.2% (-20.9 to 23.4)	0.91	1.8% (-18.5 to 27.4)	0.88
Age of mother at delivery (>30 years)	3.0% (-18.5 to 30.2)	0.80	6.1% (-16.1 to 34.2)	0.62
Alcohol consumption of mother during pregnancy	-	-	-16.0% (-31.5 to 3.1)	0.10
High blood pressure mother	-	-	53.9% (-3.7 to 146.0)	0.07
Pre-term birth	-	-	-2.7% (-25.8 to 27.9)	0.85

Estimates were presented as % difference (95% CI) in mtDNA content.

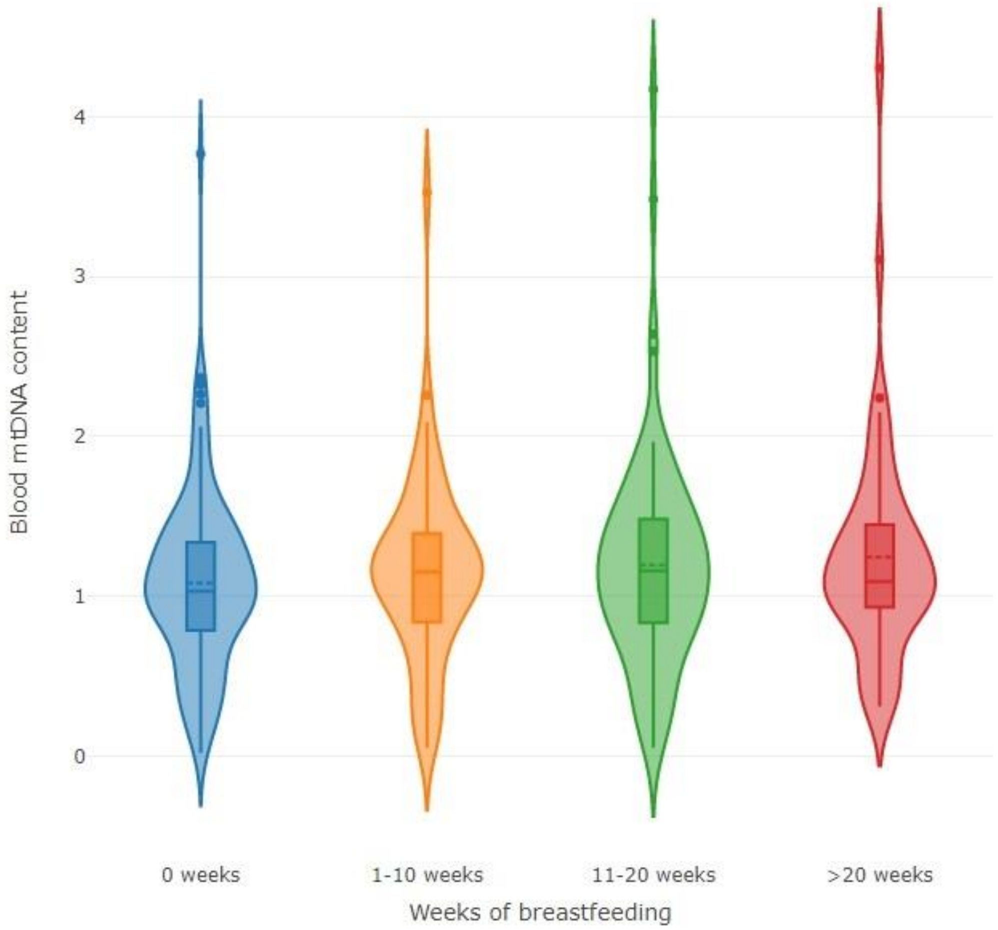

Supplementary Figure S1: Violin plot of mtDNA content in groups based on the duration of breastfeeding.

REFERENCES

1. Janssen BG, Munters E, Pieters N, Smeets K, Cox B, Cuypers A, et al. Placental mitochondrial DNA content and particulate air pollution during in utero life. Environ Health Perspect. 2012;120(9):1346-52.

2. Hellemans J, Mortier G, De Paepe A, Speleman F, Vandesompele J. qBase relative quantification framework and software for management and automated analysis of real-time quantitative PCR data. Genome Biol. 8. London2007. p. R19.
